# Poly[diaqua­(μ_3_-1*H*-benzimidazole-5,6-dicarboxyl­ato-κ^4^ *N* ^3^:*O* ^5^,*O* ^6^:*O* ^6′^)magnesium(II)]

**DOI:** 10.1107/S1600536810001029

**Published:** 2010-01-13

**Authors:** Hao Wang, Xiao-Fei Li, Wen-Dong Song, Xiao-Tian Ma, Juan-Hua Liu

**Affiliations:** a College of Food Science and Technology, Guang Dong Ocean University, Zhanjiang 524088, People’s Republic of China; b College of Agriculture, Guang Dong Ocean University, Zhanjiang 524088, People’s Republic of China; c College of Science, Guang Dong Ocean University, Zhanjiang 524088, People’s Republic of China

## Abstract

In the title complex, [Mg(C_9_H_4_N_2_O_4_)(H_2_O)_2_]_*n*_, the Mg^II^ atom is six-coordinated by one N and three O atoms from three different 1*H*-benzimidazole-5,6-dicarboxyl­ate ligands and two O atoms from two water mol­ecules, forming a slightly distorted octa­hedral geometry. The ligand links the Mg^II^ centres into a three-dimensional network. Extensive N—H⋯O and O—H⋯O hydrogen bonds exist between the ligands and water mol­ecules, stabilizing the crystal structure.

## Related literature

For related structures of 1*H*-benzimidazole-5,6-dicarboxyl­ate complexes, see: Song, Wang, Hu *et al.* (2009 ▶); Song, Wang, Li *et al.* (2009 ▶); Song, Wang, Qin *et al.* (2009 ▶); Wang *et al.* (2009 ▶).
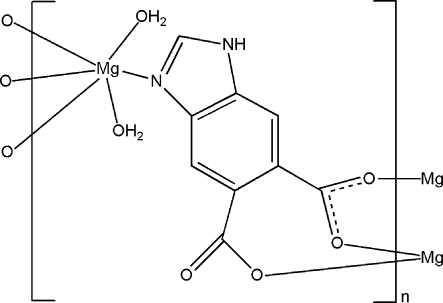


## Experimental

### 

#### Crystal data

[Mg(C_9_H_4_N_2_O_4_)(H_2_O)_2_]
*M* *_r_* = 264.48
Monoclinic, 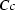

*a* = 7.4793 (15) Å
*b* = 18.958 (4) Å
*c* = 7.3132 (15) Å
β = 99.38 (3)°
*V* = 1023.1 (4) Å^3^
*Z* = 4
Mo *K*α radiation
μ = 0.20 mm^−1^
*T* = 293 K
0.30 × 0.25 × 0.21 mm

#### Data collection

Rigaku/MSC Mercury CCD diffractometer
Absorption correction: multi-scan (*REQAB*; Jacobson, 1998 ▶) *T*_min_ = 0.943, *T*_max_ = 0.960
4611 measured reflections
1143 independent reflections
1096 reflections with *I* > 2σ(*I*)
*R*_int_ = 0.031

#### Refinement

*R*[*F* ^2^ > 2σ(*F* ^2^)] = 0.036
*wR*(*F* ^2^) = 0.090
*S* = 1.06
1143 reflections
163 parameters
2 restraints
H-atom parameters constrained
Δρ_max_ = 0.38 e Å^−3^
Δρ_min_ = −0.25 e Å^−3^

### 

Data collection: *CrystalStructure* (Rigaku/MSC, 2002 ▶); cell refinement: *CrystalStructure*; data reduction: *CrystalStructure*; program(s) used to solve structure: *SHELXS97* (Sheldrick, 2008 ▶); program(s) used to refine structure: *SHELXL97* (Sheldrick, 2008 ▶); molecular graphics: *ORTEPII* (Johnson, 1976 ▶) and *DIAMOND* (Brandenburg, 1999 ▶); software used to prepare material for publication: *SHELXL97*.

## Supplementary Material

Crystal structure: contains datablocks I, global. DOI: 10.1107/S1600536810001029/hy2269sup1.cif

Structure factors: contains datablocks I. DOI: 10.1107/S1600536810001029/hy2269Isup2.hkl

Additional supplementary materials:  crystallographic information; 3D view; checkCIF report

## Figures and Tables

**Table 1:** Selected bond lengths (Å)

Mg1—N1	2.195 (3)
Mg1—O1^i^	2.051 (3)
Mg1—O3^i^	2.106 (3)
Mg1—O4^ii^	2.113 (3)
Mg1—O1*W*	2.063 (3)
Mg1—O2*W*	2.074 (3)

Symmetry codes: (i) 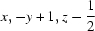; (ii) 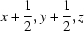.

**Table 2:** Hydrogen-bond geometry (Å, °)

*D*—H⋯*A*	*D*—H	H⋯*A*	*D*⋯*A*	*D*—H⋯*A*
N3—H3⋯O1^iii^	0.86	2.29	2.992 (4)	138
O1*W*—H1*W*⋯O2^iv^	0.84	1.84	2.651 (4)	163
O1*W*—H2*W*⋯O3^ii^	0.84	1.92	2.734 (4)	164
O2*W*—H3*W*⋯O4^iv^	0.84	2.27	3.068 (4)	160
O2*W*—H4*W*⋯O2^v^	0.84	1.87	2.685 (4)	164

Symmetry codes: (ii) 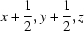; (iii) 
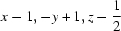
; (iv) 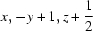; (v) 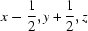.

**Fig. 1.**
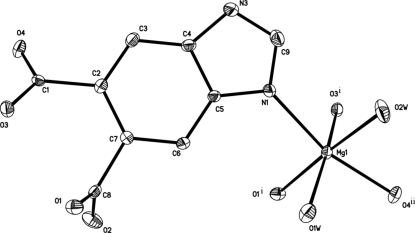
The structure of the title compound, showing the 30% probability displacement ellipsoids. [Symmetry codes: (i) x, 1 - y, -1/2 + z; (ii) 1/2 + x, 1/2 + y, z.]

**Fig. 2.**
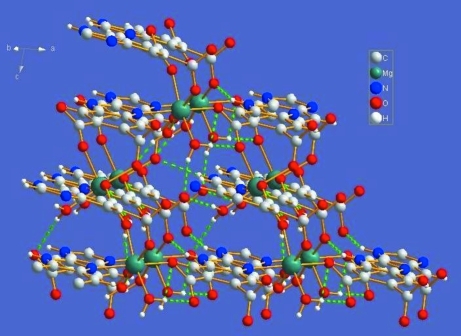
A view of the three-dimensional network structure of the title compound. Hydrogen bonds are shown as dashed lines.

## supplementary crystallographic information

### Comment

1*H*-Benzimidazole-5,6-dicarboxylic acid (H_2_*L*) can function as a multidentate ligand and several complexes formed from this ligand have been reported recently, including *catena*-poly[[diaqua(1,10-phenanthroline-κ^2^*N*,*N*') nickel(II)]-µ-*L*-κ^2^*N*^3^:*O*^6^] (Song, Wang, Hu *et al.*, 2009), pentaaqua(*L*-κ*N*^3^)cobalt(II) pentahydrate (Song, Wang, Li *et al.*, 2009), pentaaqua(*L*-κ*N*^3^)nickel(II) pentahydrate (Song, Wang, Qin *et al.*, 2009) and tetraaquabis(*L*-κ*N*^3^)cobalt(II) dimethylformamide disolvate dihydrate (Wang *et al.*, 2009). However, the Mg complex of the H_2_*L* ligand has not been reported up to now.

As shown in Fig. 1, the Mg^II^ atom is six-coordinated by one N and three O atoms from three different *L* ligands, and two O atoms from two water molecules (Table 1), showing a slightly distorted octahedral geometry. The equatorial plane is defined by O1W, O2W, O1^i^ and O3^i^ atoms, while N1 and O4^ii^ occupy the axial positions [symmetry codes: (i) *x*, 1 - *y*, -1/2 + *z*; (ii) 1/2 + *x*, 1/2 + *y*, *z*]. Intermolecular O—H···O and N—H···O hydrogen bonds between the ligand and the coordinated water molecules stabilize the structure (Table 2 and Fig 2).

### Experimental

A mixture of MgCl_2_ (1.0 mmol), H_2_*L* (0.6 mmol), CH_3_CN (6 ml) and water (4 ml) was added to a 20 ml Teflon-lined stainless container, which was heated to 150°C and held at that temperature for 5 d. After cooling to room temperature, colourless crystals were recovered by filtration.

### Refinement

C– and N-bound H atoms were placed at calculated positions and treated as riding on the parent C or N atoms, with C—H = 0.93 and N—H = 0.86 Å, and with *U*_iso_(H) = 1.2*U*_eq_(C, N). The water H atoms were located in a difference Fourier map and refined as riding with a distance restraint of O—H = 0.84 Å and with *U*_iso_(H) = 1.5*U*_eq_(O).

### Crystal data

**Table:**

[Mg(C_9_H_4_N_2_O_4_)(H_2_O)_2_]	*F*(000) = 544
*M**_r_* = 264.48	*D*_x_ = 1.717 Mg m^−^^3^
Monoclinic, *C**c*	Mo *K*α radiation, λ = 0.71073 Å
Hall symbol: C -2yc	Cell parameters from 4114 reflections
*a* = 7.4793 (15) Å	θ = 3.6–27.5°
*b* = 18.958 (4) Å	µ = 0.20 mm^−^^1^
*c* = 7.3132 (15) Å	*T* = 293 K
β = 99.38 (3)°	Block, colourless
*V* = 1023.1 (4) Å^3^	0.30 × 0.25 × 0.21 mm
*Z* = 4	

### Data collection

**Table:**

Rigaku/MSC Mercury CCD diffractometer	1143 independent reflections
Radiation source: fine-focus sealed tube	1096 reflections with *I* > 2σ(*I*)
graphite	*R*_int_ = 0.031
ω scans	θ_max_ = 27.5°, θ_min_ = 3.6°
Absorption correction: multi-scan (REQAB; Jacobson, 1998)	*h* = −9→9
*T*_min_ = 0.943, *T*_max_ = 0.960	*k* = −24→24
4611 measured reflections	*l* = −8→9

### Refinement

**Table:**

Refinement on *F*^2^	Primary atom site location: structure-invariant direct methods
Least-squares matrix: full	Secondary atom site location: difference Fourier map
*R*[*F*^2^ > 2σ(*F*^2^)] = 0.036	Hydrogen site location: inferred from neighbouring sites
*wR*(*F*^2^) = 0.090	H-atom parameters constrained
*S* = 1.06	*w* = 1/[σ^2^(*F*_o_^2^) + (0.04*P*)^2^ + 2*P*] where *P* = (*F*_o_^2^ + 2*F*_c_^2^)/3
1143 reflections	(Δ/σ)_max_ < 0.001
163 parameters	Δρ_max_ = 0.38 e Å^−^^3^
2 restraints	Δρ_min_ = −0.25 e Å^−^^3^

### Fractional atomic coordinates and isotropic or equivalent isotropic displacement parameters (Å^2^)

**Table:**

	*x*	*y*	*z*	*U*_iso_*/*U*_eq_	
Mg1	0.27807 (16)	0.68928 (5)	0.63241 (16)	0.0172 (3)	
O1	0.4349 (4)	0.37147 (14)	0.9888 (4)	0.0234 (5)	
O2	0.5047 (4)	0.37926 (16)	0.7087 (4)	0.0317 (7)	
O3	0.1320 (4)	0.28489 (12)	0.8699 (4)	0.0237 (6)	
O4	−0.0733 (4)	0.28304 (12)	0.6132 (3)	0.0236 (6)	
N1	0.0678 (5)	0.61142 (15)	0.6633 (5)	0.0214 (6)	
N3	−0.2035 (5)	0.56134 (16)	0.6122 (5)	0.0271 (7)	
H3	−0.3196	0.5577	0.5874	0.033*	
C1	0.0380 (5)	0.31481 (16)	0.7326 (5)	0.0173 (6)	
C2	0.0549 (5)	0.39389 (16)	0.7123 (5)	0.0182 (7)	
C3	−0.1002 (5)	0.43318 (18)	0.6560 (5)	0.0223 (7)	
H2	−0.2127	0.4116	0.6240	0.027*	
C4	−0.0816 (5)	0.50629 (18)	0.6490 (5)	0.0206 (7)	
C5	0.0876 (5)	0.53907 (17)	0.6828 (5)	0.0184 (7)	
C6	0.2438 (5)	0.49930 (18)	0.7332 (5)	0.0185 (7)	
H1	0.3574	0.5206	0.7531	0.022*	
C7	0.2259 (4)	0.42738 (16)	0.7529 (4)	0.0154 (6)	
C8	0.3985 (5)	0.38839 (17)	0.8204 (5)	0.0173 (7)	
C9	−0.1073 (6)	0.62155 (18)	0.6224 (6)	0.0262 (8)	
H9	−0.1599	0.6659	0.6023	0.031*	
O1W	0.4663 (4)	0.65596 (14)	0.8537 (4)	0.0289 (6)	
H1W	0.4573	0.6408	0.9598	0.043*	
H2W	0.5355	0.6911	0.8601	0.043*	
O2W	0.1388 (5)	0.75021 (15)	0.7982 (5)	0.0374 (8)	
H3W	0.0660	0.7342	0.8639	0.056*	
H4W	0.1176	0.7927	0.7704	0.056*	

### Atomic displacement parameters (Å^2^)

**Table:**

	*U*^11^	*U*^22^	*U*^33^	*U*^12^	*U*^13^	*U*^23^
Mg1	0.0191 (6)	0.0134 (4)	0.0184 (5)	−0.0004 (4)	0.0009 (4)	0.0004 (4)
O1	0.0186 (13)	0.0310 (13)	0.0202 (12)	0.0004 (10)	0.0019 (11)	0.0074 (10)
O2	0.0247 (16)	0.0462 (17)	0.0253 (14)	0.0133 (12)	0.0075 (12)	0.0005 (12)
O3	0.0274 (14)	0.0165 (11)	0.0235 (12)	−0.0008 (10)	−0.0074 (11)	0.0006 (9)
O4	0.0281 (15)	0.0195 (12)	0.0203 (13)	−0.0067 (10)	−0.0045 (11)	0.0014 (9)
N1	0.0215 (16)	0.0167 (13)	0.0265 (15)	0.0018 (11)	0.0058 (12)	0.0039 (11)
N3	0.0147 (16)	0.0249 (14)	0.0400 (19)	0.0032 (12)	−0.0009 (14)	0.0065 (13)
C1	0.0195 (17)	0.0151 (13)	0.0175 (16)	−0.0016 (12)	0.0043 (14)	−0.0002 (11)
C2	0.0197 (18)	0.0150 (14)	0.0192 (16)	−0.0024 (12)	0.0008 (14)	0.0015 (12)
C3	0.0184 (18)	0.0204 (16)	0.0274 (19)	−0.0040 (13)	0.0011 (15)	0.0023 (13)
C4	0.0151 (19)	0.0216 (16)	0.0244 (18)	0.0023 (12)	0.0011 (14)	0.0045 (13)
C5	0.0191 (17)	0.0160 (14)	0.0203 (16)	−0.0019 (12)	0.0035 (14)	0.0016 (12)
C6	0.0129 (16)	0.0198 (15)	0.0224 (17)	−0.0022 (11)	0.0018 (14)	0.0008 (12)
C7	0.0151 (16)	0.0178 (14)	0.0134 (14)	0.0002 (12)	0.0027 (13)	−0.0002 (11)
C8	0.0154 (17)	0.0154 (14)	0.0204 (17)	−0.0024 (11)	0.0007 (14)	−0.0011 (11)
C9	0.024 (2)	0.0208 (16)	0.034 (2)	0.0068 (14)	0.0050 (17)	0.0067 (14)
O1W	0.0340 (15)	0.0271 (13)	0.0225 (12)	−0.0071 (11)	−0.0043 (12)	0.0052 (10)
O2W	0.0486 (19)	0.0219 (12)	0.0476 (19)	0.0063 (12)	0.0251 (16)	−0.0022 (12)

### Geometric parameters (Å, °)

**Table:**

Mg1—N1	2.195 (3)	C1—C2	1.514 (4)
Mg1—O1^i^	2.051 (3)	C2—C3	1.384 (5)
Mg1—O3^i^	2.106 (3)	C2—C7	1.416 (5)
Mg1—O4^ii^	2.113 (3)	C3—C4	1.395 (5)
Mg1—O1W	2.063 (3)	C3—H2	0.9300
Mg1—O2W	2.074 (3)	C4—C5	1.395 (5)
O1—C8	1.259 (4)	C5—C6	1.388 (5)
O2—C8	1.241 (4)	C6—C7	1.380 (5)
O3—C1	1.263 (4)	C6—H1	0.9300
O4—C1	1.257 (4)	C7—C8	1.499 (5)
N1—C9	1.310 (5)	C9—H9	0.9300
N1—C5	1.384 (4)	O1W—H1W	0.8401
N3—C9	1.345 (5)	O1W—H2W	0.8400
N3—C4	1.383 (5)	O2W—H3W	0.8399
N3—H3	0.8600	O2W—H4W	0.8400
			
O1^i^—Mg1—O1W	81.69 (12)	C7—C2—C1	120.7 (3)
O1^i^—Mg1—O2W	174.71 (15)	C2—C3—C4	117.5 (3)
O1W—Mg1—O2W	93.18 (14)	C2—C3—H2	121.2
O1^i^—Mg1—O3^i^	85.34 (12)	C4—C3—H2	121.3
O1W—Mg1—O3^i^	166.51 (13)	N3—C4—C3	133.6 (3)
O2W—Mg1—O3^i^	99.68 (13)	N3—C4—C5	104.4 (3)
O1^i^—Mg1—O4^ii^	95.02 (12)	C3—C4—C5	122.0 (3)
O1W—Mg1—O4^ii^	90.61 (11)	N1—C5—C6	129.5 (3)
O2W—Mg1—O4^ii^	83.68 (12)	N1—C5—C4	110.1 (3)
O3^i^—Mg1—O4^ii^	86.80 (11)	C6—C5—C4	120.3 (3)
O1^i^—Mg1—N1	98.87 (12)	C7—C6—C5	118.2 (3)
O1W—Mg1—N1	97.00 (12)	C7—C6—H1	120.9
O2W—Mg1—N1	82.98 (13)	C5—C6—H1	120.9
O3^i^—Mg1—N1	88.65 (12)	C6—C7—C2	121.4 (3)
O4^ii^—Mg1—N1	164.98 (12)	C6—C7—C8	115.4 (3)
C8—O1—Mg1^iii^	126.4 (2)	C2—C7—C8	123.2 (3)
C1—O3—Mg1^iii^	139.4 (2)	O2—C8—O1	123.3 (3)
C1—O4—Mg1^iv^	130.8 (2)	O2—C8—C7	117.5 (3)
C9—N1—C5	104.8 (3)	O1—C8—C7	119.0 (3)
C9—N1—Mg1	125.8 (2)	N1—C9—N3	113.2 (3)
C5—N1—Mg1	127.6 (3)	N1—C9—H9	123.4
C9—N3—C4	107.4 (3)	N3—C9—H9	123.4
C9—N3—H3	126.3	Mg1—O1W—H1W	133.0
C4—N3—H3	126.3	Mg1—O1W—H2W	97.9
O4—C1—O3	123.8 (3)	H1W—O1W—H2W	111.1
O4—C1—C2	117.6 (3)	Mg1—O2W—H3W	124.6
O3—C1—C2	118.6 (3)	Mg1—O2W—H4W	119.1
C3—C2—C7	120.4 (3)	H3W—O2W—H4W	111.8
C3—C2—C1	118.9 (3)		

Symmetry codes: (i) *x*, −*y*+1, *z*−1/2; (ii) *x*+1/2, *y*+1/2, *z*; (iii) *x*, −*y*+1, *z*+1/2; (iv) *x*−1/2, *y*−1/2, *z*.

### Hydrogen-bond geometry (Å, °)

**Table:**

*D*—H···*A*	*D*—H	H···*A*	*D*···*A*	*D*—H···*A*
N3—H3···O1^v^	0.86	2.29	2.992 (4)	138
O1W—H1W···O2^iii^	0.84	1.84	2.651 (4)	163
O1W—H2W···O3^ii^	0.84	1.92	2.734 (4)	164
O2W—H3W···O4^iii^	0.84	2.27	3.068 (4)	160
O2W—H4W···O2^vi^	0.84	1.87	2.685 (4)	164

Symmetry codes: (v) *x*−1, −*y*+1, *z*−1/2; (iii) *x*, −*y*+1, *z*+1/2; (ii) *x*+1/2, *y*+1/2, *z*; (vi) *x*−1/2, *y*+1/2, *z*.
